# Measuring Learner Satisfaction of an Adaptive Learning System

**DOI:** 10.3390/bs12080264

**Published:** 2022-07-30

**Authors:** Lyndon Lim, Seo Hong Lim, Rebekah Wei Ying Lim

**Affiliations:** Teaching & Learning Centre, Singapore University of Social Sciences, Singapore 599494, Singapore; shlim@suss.edu.sg (S.H.L.); rebekahlimwy@suss.edu.sg (R.W.Y.L.)

**Keywords:** learner satisfaction, tertiary education, validation, measurement

## Abstract

Tertiary educational institutions have continually implemented various educational technologies to support student learning. An example is adaptive learning systems, within which learners take ownership of their learning experience and accelerate future learning. Despite the importance of considering learner satisfaction in the development of such systems given how it has been widely acknowledged as an indication of the success of e-learning systems, research in the area of adaptive learning technologies for education has concentrated more on tailoring instruction to implement personalised learning. A review of instruments measuring learner satisfaction of virtual learning environments found that the learner satisfaction questionnaire (LSQ) that was developed to measure learner satisfaction of e-learning systems, and preliminarily validated by means of exploratory factor analysis, was most suitable for adaptation. This study sought to adapt and validate the LSQ for the purpose of measuring learner satisfaction of an adaptive learning system developed in-house. A total of 121 tertiary students recruited via availability sampling took part in this study. Hierarchical confirmatory factor analysis was performed as part of the validation. Results presented the adapted LSQ as a 14-item instrument that can be readily deployed on a broad scale basis. The adapted LSQ also yielded valid and reliable satisfaction scores both at the subscale as well as the overall scale level. Practical implications are discussed, noting that such scores could inform the further development and refinement of AdLeS or similar systems, with the view of benefiting students.

## 1. Introduction

To enhance teaching and learning, tertiary educational institutions have implemented various technologies for learners of diverse backgrounds. Further, due to the prevalence of continuing and further education, adult learners are increasingly enrolled in multiple courses with components of online delivery. However, after being away from the classroom for some time, adult learners might encounter learning roadblocks and subsequently experience difficulties in learning [1]. According to the theory of adult learning in andragogy [2], adult learners learn more effectively in a self-directed learning context rather than a teacher-centred classroom, akin to the traditional pedagogical approach [3]. Given this, a student-centred context for adult learners is essential as it focuses on learning experience [4], individual interest, and autonomy; in doing so, a student-centred context supports understanding and deep learning [5,6]. To promote student-centred contexts, adaptive learning systems have been merged with conventional didactic lectures to enhance active learning via blended learning [7]; adaptive learning systems can assist learners and provide them a dynamic learning experience capable of enhancing satisfaction, motivation, and potentially positive learning outcomes via facilitating the personalisation of educational activities [8].

With adaptive learning systems, learners could take ownership of their learning experience [9] and accelerate future learning [10]. However, there are challenges with online technologies such as unsatisfying interactions [11]. When analysing user satisfaction and system quality, Ref. [12] stated that the former is central to the success of an information system. In agreement with Ref. [12], researchers have further added that satisfaction and self-efficacy lead to user intentions of utilising learning systems [13,14]. Even though it is crucial to account for user satisfaction as it affects learning, research in the area of adaptive learning technologies for education has concentrated more on tailoring instruction to implement personalised learning, rather than exploring how personalised learning is related to learner satisfaction [15]. Further, multiple published instruments assessing learner satisfaction have been developed within the context of instructor-led courses, as opposed to student-centred, self-led adaptive learning courses. Given these, it is worthwhile to present an instrument ready for practical use to assess learner satisfaction in adaptive learning systems; this study seeks to adapt and validate an instrument for the purpose of examining learner satisfaction in relation to the use of an adaptive learning system (AdLeS) developed in-house by a tertiary educational institution.

### 1.1. Learner Satisfaction

In technology-enhanced learning environments, learner satisfaction has been widely acknowledged as an indication of the success of e-learning systems [16,17,18]. Due to its direct and significant relationship with overall system use, user satisfaction is known as an imperative factor for evaluating the success of an information system [19,20,21]. The empirical research of [22] reported that user satisfaction and acceptance should be considered when researching users’ perceptions on any information system. Similarly, Refs. [23,24] concurred with similar viewpoints by postulating that user satisfaction is one of the most important factors when addressing the success of an information system. Nonetheless, this has not been thoroughly evident within research of adaptive learning technologies for education, with more focussing on methods to personalise learning [15].

Learner satisfaction is defined as a user’s perception of a particular information system that can be useful and effective for achieving one’s objectives [25]. It refers to a learner’s personal feelings about the learning process in which satisfaction and positive feelings are generated and experienced during learning activities and reflects how students perceive their learning experiences associated with programme quality, evaluation, and student-related outcomes [26,27]. High user satisfaction contributes to lower drop-out rates, higher persistence, and commitment to a programme [27,28,29].

Learner satisfaction plays a significant role due to the relationship between users and e-learning systems [30], and hence has to be considered for the purposes relevant to the quality of newly developed e-learning environments. Essentially, if learners are more satisfied with a particular system, their intention to continue using it would be proportionately enhanced [17]. Researchers have indicated that success of an e-learning system can be measured with learners’ satisfaction and intention to adopt it for a longer period of time [31]. Further, Refs. [32,33] highlighted the use of users’ continued satisfaction as an indication of success of an e-learning system. In this regard, Ref. [34] pointed out that, to assess the long-term applications of information systems, it is crucial to measure learner satisfaction. In particular, learner satisfaction is essential in assisting relevant stakeholders in understanding system functionalities, performances, and capabilities to facilitating a productive learning experience for its users. The understanding of learner satisfaction helps to elucidate multidimensional factors within e-learning such as technology support, pedagogical contents, instruction, and feedback.

### 1.2. Antecedents of Learner Satisfaction

To investigate the potential factors influencing learner satisfaction and academic achievement when utilising an online learning platform in higher education, Ref. [35] found that student background, experience, collaboration, interaction, and autonomy affected student satisfaction. Similarly, to identify whether technology integration enhances learner satisfaction, improves academic performance, and achieves continued intention to use, Ref. [36] found that learner satisfaction and academic performance could affect continued intention to use e-learning systems. Further, Ref. [37] pointed out that different applications of technologies in higher education could directly or indirectly interfere with learner academic performance and satisfaction; this suggests that existing learner satisfaction measures for established e-learning systems might be less applicable for novel adaptive learning systems (e.g., AdLeS). In addition to the studies discussed, Table 1 provides a broader view of research of key antecedents contributing to learner satisfaction within the context of e-learning.

### 1.3. Measures of Learner Satisfaction

Within the literature, the measurement of learner satisfaction in e-learning systems has been presented in multiple forms by means of modelling the construct with different sub-scales (see Table 1). As an example, Ref. [42] concluded, on the basis of a 132-item questionnaire, that learner satisfaction is a construct with six sub-scales (i.e., learner dimension, instructor’s dimension, course dimension, technology dimension, design dimension, and the environment dimension). In a latter work within a different context, Ref. [40] proposed that learner satisfaction could be measured via five subscales (i.e., perceived usefulness, course quality, system quality, instructor quality, and service quality) on the basis of a 25-item questionnaire. While the measures in Table 1 attempted to address learner satisfaction, some presented subscales were associated with the computer-based assessment acceptance model (CBAAM) proffered by [48] (e.g., [36,40]). It should be noted that, rather than a measure of learner satisfaction, the CBAAM was developed to explain the intention to use computer-based assessment and was a continuation of work completed to ascertain the acceptance of assessment within learning management systems [49]. To this end, some of the existing measures might be less appropriate for determining learner satisfaction of e-learning systems.

Noting the multiple representations of learner satisfaction and the feasibility of including a purposive questionnaire to assess learner satisfaction (i.e., for AdLeS), the review undertaken in this study found that the instrument developed by [47] applicable for adaptation. A key reason for suitability is the omission of sub-scales present in some of the existing learner satisfaction measures that AdLeS, being student-centred, would not provide (e.g., instructor quality, service quality, collaborative learning). The empirical work and learner satisfaction measurement model of [47] resulted in a 17-item learner satisfaction questionnaire (LSQ) with four sub-scales (i.e., learner interface, learning community, content, and personalisation) that measure learner satisfaction toward e-learning systems. Further, the learner satisfaction model by [47] has been validated in an Asian context similar to the one within this study (i.e., adult learners in an Asian country).

While not all had been related to adaptive learning systems, the studies in Table 1 provide an understanding of antecedents of learner satisfaction in relation to the utilisation of e-learning technologies. Evidently, most of the studies included the dimension of instructor presence and support or collaboration, dimensions adaptive learning systems do not normally afford. Further, in some of these studies, learner satisfaction had been measured via one subscale (e.g., four items), presenting and over-simplifying satisfaction as a non-complex and unidimensional construct. Given these points and the fact that some of these subscales have not been validated psychometrically, there is a need for more published research concerning learner satisfaction in adaptive learning systems where the instructor is not present. This study aims to contribute to scholarship related to learner satisfaction in adaptive learning systems.

## 2. Methodology

Two stages were undertaken in this study, according to validity evidence based on content appropriateness and that based on internal structure as recommended in the Standards for Educational and Psychological Testing [50]. Stage 1 involved determining content appropriateness of the LSQ so that the items would be applicable to the intended sample (i.e., part-time undergraduates reading Calculus via AdLeS) within the context of this study. Following stage 1, the LSQ-adapted (LSQ_a_) was administered to participants recruited on the basis of availability sampling [51]; ethics approval was sought from the university institution review board before participants were asked to complete the LSQ_a_ voluntarily. The study then proceeded with Stage 2 that was based upon the empirical work by [47] who found that learner satisfaction toward e-learning systems presented by the LSQ could be explained by four subscales (i.e., learner interface, learning community, content, personalisation). As the work of [47] was limited to exploratory factor analysis and that the LSQ was adapted for this study, a confirmatory factor analysis (CFA) was required [52]. Hence, a hierarchical CFA was performed to ascertain the extent of how well the pre-specified learner satisfaction measurement model by [47] (see Appendix A) fitted the sample within this study. An acceptable fit of the sample data to the model would provide evidence to support valid learner satisfaction scores which could then be used to inform further developments of AdLeS.

### 2.1. Participants

Participants comprised 81.2% of the part-time undergraduates enrolled in a level-one Calculus course across one semester in a tertiary educational institution (see Table 2) in Singapore. Their age ranged between 20 and 67 years (M = 27.68, SD = 7.02 years), and 22.2% identified themselves as female. There were 121 participants in total, and this was deemed adequate on the basis of the recommendations of [53,54], and considering the simplicity of the pre-specified measurement model proposed by [47] (see Appendix A).

### 2.2. Stage 1

In this stage, all 17 items of the original LSQ were reviewed for content appropriateness by two academics (i.e., the course director and one senior lecturer) and a senior research associate involved in the Calculus course and the development of AdLeS in the tertiary educational institution. Given that the LSQ was preliminarily validated by [47] on a sample comprising adult learners in Taiwan, the items were found to be suitable for use for the intended sample within this study. There were exceptions of some minor edits, the exclusion of the *learning community* dimension, and the addition of two items (see Appendix B). The *learning community* dimension was excluded as AdLeS was intended to be student-centred and self-paced, and did not offer avenues for collaboration or discussion with other students. On the contrary, the addition of two items (i.e., the system supports my learning; the system recommends topics that reflects my learning progress) reflected the intent of AdLeS. The review in stage 1 led to the development of the LSQ_a_, which was subsequently administered to the participants after they completed the course via AdLeS.

### 2.3. AdLeS

AdLeS is a prototype developed to support the diverse learning needs of adult part-time undergraduates enrolled in some courses (e.g., foundational Calculus) within the tertiary educational institution in this study. The system behind AdLeS was guided by the elements of adaptivity (i.e., testing, content and sequencing) and developed on the basis of algorithms intended to gather data in real time while students interacted with it. On the basis of these interactions, items and content deemed suitable for the student would be presented accordingly. For example, a low-progress student would be able to access item hints and content with more examples, as compared with a high-progress student. In this regard, the intent of AdLeS is to provide learning pathways suitable and more efficient for each student according to their strengths and weaknesses.

### 2.4. Stage 2

In this stage, the covariance analysis of linear structural equations procedure in SAS (9.4) was used to conduct hierarchical CFA. CFA served to assess the dimensionality of the LSQ_a_ and determine the appropriateness of the factorial structure established through exploratory factor analysis proffered by [47].

Normality tests were performed prior to further analyses in order to ascertain the appropriateness of using CFA in this study. Tests of univariate normality (i.e., Shapiro–Wilk, Kolmogorov–Smirnov, Cramer–von Mises, and Anderson–Darling) and Mardia tests of multivariate normality showed that all 14 items had non-normal distributions. Nonetheless, the data were considered appropriate for CFA, as the skewness and kurtosis of all the items (see Appendix C) were within the recommendations required for structural equation modelling by [55] (i.e., skewness and kurtosis indices should be within ±2 and ±7 correspondingly).

Following this, a hierarchical CFA approach was undertaken for this study to ascertain the three-factor second-order measurement model of the LSQ pre-specified by [47]; one-factor, correlated three-factor, second-order three-factor, and bifactor three-factor models were examined. Confirmatory factor models within this study were evaluated on the basis of recommendations by [52,56,57]. As the sample presented modest non-normalities, the maximum likelihood with Satorra–Bentler scaled chi-squared statistic for model fit that adjusts the chi-squared statistic and standard errors for data non-normality was used for more precise goodness-of-fit statistics [49,58].

## 3. Results

Table 3 presents the results of the hierarchical CFA. Clearly, the one-factor model did not present psychometrically sound evidence, and hence, it would not be tenable to total and average LSQ_a_ item scores to represent a single dimension of learner satisfaction toward AdLeS. The three-factor first-order model appeared plausible though items LI3 and PERS3 returned standardised loadings below the recommended threshold of 0.7 [52] (see Table 4). Further, discriminant validity issues remained as presented by the average variance extracted for each factor (see Table 5), consistent with recommendations by [52] (e.g., the item measures of the subscale CONT did not appear to explain more of the variance than it shared with the PERS and LI subscales). Owing to this, a three-factor second-order CFA was performed restricting correlation between factors; performing a second-order CFA was also considerably more viable when the first-order CFA returned discriminant validity issues [59]. This subsequent CFA found fit indices identical to the prior model. As with most hierarchical CFA, a bifactor analysis was further undertaken, though the results did not favour the rejection of the second-order model.

On the basis of the four competing models presented in Table 3 and considering the discriminant validity issue with the three-factor first-order model, the three-factor second-order model was concluded as most plausible.

In the three-factor second-order model, all standardised loading estimates were significant (*p* < 0.001) and 0.7 or greater except for item PERS4 (0.59) (see Table 6) that was retained considering the modest departure from 0.7, on the basis that a significant loading 0.5 or greater should be considered for practical significance [52]. This retention is also tenable given that intent of item PERS4 (i.e., the system recommends topics that reflects my learning progress) is to gather information with regard to the degree of how much students perceive AdLeS to recommend suitable topics as they engage with it; the more students perceive that AdLeS recommends topics that reflect their learning progress, the more they would be satisfied with the system. The intent of item PERS4 is also consistent with that of AdLeS and adaptive learning systems in general, that is, to present items and content deemed suitable for the student on the basis of how they interact with the system [60]. Further to the standardised loadings, the average variance extracted and construct reliability coefficients were above 0.5 and 0.7, respectively, suggesting adequate reliability and acceptable error variance [52].

## 4. Discussion and Directions for Future Research

Results from stages 1 and 2 of this study suggested a three-factor second-order measurement model explaining learner satisfaction toward an in-house adaptive learning system (i.e., AdLeS). Items reviewed, retained, and added from stage 1 appeared to fit the three-factor second-order measurement model. The two additional items (i.e., CONT6—the system supports my learning, and PERS4—the system recommends topics that reflects my learning progress) were clustered within the CONT and PERS subscales, respectively. This is within expectation, as item CONT6 is related to how students perceive AdLeS as a support to their learning, and that content in AdLeS inevitably plays a role in this support; item PERS4 is related to how students perceive AdLeS to be adaptive, as reflected by how AdLeS recommends topics that reflect students’ learning.

Given the fit of the data to the three-factor second-order measurement model, it would be tenable to average the subscale scores to represent each of the subscales. Further, the three subscale scores could be totalled and averaged to present a learner satisfaction toward AdLeS score. On the basis of these, the LSQ_a_ holds promise as an instrument that can provide valid information at the subscale (i.e., content, personalisation, and learner interface) and overall scale level (learner satisfaction), particularly as part of the development works of adaptive learning systems.

Potential users of the LSQ_a_ should note that it had been validated on the basis of a sample of adult part-time undergraduates in Singapore. If there are reasons to suggest that the characteristics of a sample is drastically different from that in this study, a validation based on that sample should be undertaken. After all, the “validation process never ends, as there is always additional information that can be gathered to more fully understand a test and the inferences that can be drawn from it” [50]. Further, it is noteworthy that the LSQ_a_ was adapted and refined on the basis of the affordances of the in-house adaptive learning system, AdLeS. While AdLeS was designed with the common elements of adaptive learning recommended by [60], a system that affords more than these common elements should correspond with a modified LSQ_a_ to reflect how students might perceive these added affordances.

## 5. Conclusions and Practical Implications

As with most adaptive learning systems, the development of AdLeS is an ongoing process, so that learner needs and satisfaction can be better met. Institutions and agents involved in these development or refinement works have the moral obligation to determine how satisfied learners are with such systems, noting that educational institutions have increasingly implemented various technologies for learners. There are existing measures to assess learner satisfaction, but as the review undertaken in this study found, none were suitable for measuring learner satisfaction of an adaptive learning system on a broad scale basis. Validation of the measures reviewed were found to be limited. Some of these instruments have more than a hundred items, making it impractical for use. Others measure learner satisfaction on the basis of a subscale comprising as few as two items; these provide but a snapshot with the assumption that learner satisfaction is non-complex and unidimensional (e.g., I would be willing to take a fully online course again). Further, some learner satisfaction measures reviewed piggyback much larger institution-wide course evaluation ratings or are anchored upon less relevant frameworks (e.g., CBAAM).

On the basis of the review, this study adapted and validated the LSQ proposed by [47]. The validation resulted in an adapted LSQ, presented as the LSQ_a_. The LSQ_a_ not only presents a 14-item instrument deemed manageable for respondents to complete within 10 minutes, but also one yields valid and reliable satisfaction scores both at the subscale as well as the overall scale level. Such scores could inform the further development and refinement of AdLeS or similar systems, with the view of benefiting students.

Beyond instrumentation, it will be prudent and beneficial for institutions to use other methods such as interviews or focus groups to elicit or unpack learner satisfaction. Such additional data will enrich the understanding of the LSQ_a_ subscales as well as the overall scale from the students’ perspectives. Additionally, suggestions on how to improve the ratings of the subscales can be solicited from students, thereby providing practicable and relevant ideas on how learning experiences can be improved.

## Figures and Tables

**Table 1:** Studies of antecedents of learner satisfaction in chronological order.

Author(s)	Participants	Antecedents of Learner Satisfaction	Learner Satisfaction Measure
[35] Abuhassna et al. (2020)	243 higher education students	- Learners’ prior background towards online platforms - Learners’ experience - Learners’ interaction with instructor throughout the learning experience - Students’ autonomy	Of the 27-item questionnaire across five subscales, one five-item subscale was used to measure learner satisfaction. Items were not reported though entire measurement model was validated by means of structural equation modelling.
[38] Bi et al. (2020)	44 students completed the activity as part of an online business management module	- Multisensorial experience - Olfaction effect - Airflow effect	Learner satisfaction was reflected by single items (e.g., 55.6% of participants satisfied with the platform used to deliver content).
[39] Salam and Farooq (2020)	120 undergraduate students using an online information-based system	- System quality - Service quality - High sociability quality	Four-item subscale (e.g., I like working with the platform; I find the platform useful for collaborative learning) out of a 48-item questionnaire was used to measure learner satisfaction. Validation of questionnaire was by means of structural equation modelling.
[33] Al-Samarraie et al. (2018)	38 postgraduate students and 9 instructors with e-learning experience	- Information quality - The fit between task and technology - System quality - Utility value - Usefulness	Meta-analysis via the Fuzzy Decision Making Trial and Evaluation Laboratory (DEMATEL) method.
[40] Mtebe and Raphael (2018)	153 students using an e-learning platform (i.e., Moodle)	- System quality - Course quality - Service quality - Instructor quality - Perceived usefulness - Learner satisfaction	25-item questionnaire across six subscales with one subscale of three items on learner satisfaction. Validation was limited to exploratory factor analysis.
[36] Tawafak et al. (2018)	295 undergraduates using an e-learning system	- Learners’ perceived usefulness - Learners’ perceived ease of use - Learners’ satisfaction affects continued intention to use	35-item questionnaire across 12 subscales with one subscale of two items on learner satisfaction. Validation of questionnaire was by means of structural equation modelling.
[41] Virtanen et al. (2017)	115 students using virtual and digital learnings	- Diverse, motivational, and clear supportive learning material - Easy to use and re-use learning content	24-item questionnaire across five subscales. Validation of questionnaire was limited to a reliability measure (i.e., Cronbach’s alpha).
[42] Asoodar et al. (2016)	600 undergraduates using an e-learning system (i.e., Moodle)	- Learner dimension - Instructor’s dimension - Course dimension - Technology dimension - Design dimension - Environment dimension	132-item questionnaire across six subscales. Validation of questionnaire was limited to principal components and parallel analyses.
[43] Kuo et al. (2014)	221 participants from undergraduate and graduate online classes	- Learner–content interaction - Learner–instructor interaction	Five-item subscale on learner satisfaction focussed on satisfaction about course (e.g., this course contributed to my educational development; in the future, I would be willing to take a fully online course again).
[44] Ladyshewsky (2013)	Post graduate participants from six online courses with class sizes averaging around 35 students	- Instructor’s presence in areas of feedback - Quality of teaching - Facilitation of productive discourse	Learner satisfaction data (11 items) was collected using the university’s standardised course evaluation system. Validation of learner satisfaction measure was not observed.
[45] Paechter et al. (2010)	2196 participants using an e-learning system	- Clarity of the course structure - Pace of learning - Opportunities for self-regulated learning and collaborative learning - Tasks results	Learner satisfaction was reflected by learner expectations and assessment of course outcomes. Validation of learner satisfaction measure was not observed.
[46] Wu et al. (2010)	212 participants from blended e-learning course	- Performance expectations - Learning climate - Learning satisfaction - Interaction - Content feature - System functionality - Computer self-efficacy	21-item questionnaire across seven subscales with one subscale of four items on learner satisfaction. Validation of questionnaire was validated via confirmatory factor analysis.
[47] Wang (2003)	116 adult learners using an e-learning system	- Learner interface - Learning community - Content - Personalisation	17-item questionnaire across four subscales. Validation of questionnaire was limited to exploratory factor analysis.

**Table 2:** Participants.

Course/Semester/Year	Number of Enrolled Students	Number of Students Who Volunteered and Completed the LSQ_a_
Calculus I/2/2021	93	80
Calculus II/2/2021	56	41

**Table 3:** CFA goodness-of-fit indicators.

Model	x^2^	x^2^*_diff_*	*df*	x^2^*/df*	CFI	RMSEA	SRMR	AIC	SBC
One-factor	160.91 *	−	77	2.09	0.87	0.10	0.08	216.91	295.19
Correlated 3-factor	111.72		74	1.74	0.94	0.07	0.07	173.72	260.39
Second-order 3-factor	111.72		74	1.74	0.94	0.07	0.07	173.72	260.39
Bifactor 3-factor	96.71		56	1.73	0.64	0.08	0.07	194.71	331.70

Note. x^2^ = chi-squared statistic; x^2^*_diff_* is computed relative to the previous non-rejected model; *df* = degrees of freedom; CFI = comparative fit index; RMSEA = root mean square error of approximation; SRMR = standardised root mean square; AIC = Akaike information criterion; SBC = Schwarz Bayesian Criterion.* *p* < 0.001.

**Table 4:** Standardised loadings, average variance extracted, and construct reliability coefficients of the three-factor first-order model.

Construct and Items	Standardised Loading	Average Variance Extracted	Construct Reliability
LI (F1)		0.64	0.98
LI1	0.85		
LI2	0.85		
LI3	0.65		
LI4	0.82		
CONT (F2)		0.61	0.99
CONT1	0.73		
CONT2	0.85		
CONT3	0.76		
CONT4	0.79		
CONT5	0.80		
CONT6	0.75		
PERS (F3)		0.54	0.97
PERS1	0.75		
PERS2	0.81		
PERS3	0.59		
PERS4	0.78		

Note. LI = learner interface; CONT = content; PERS = personalisation.

**Table 5:** Distinctiveness of sub-constructs of the three-factor first-order model.

Construct	LI	CONT	PERS
LI	0.80 *		
CONT	0.81	0.78 *	
PERS	0.66	0.81	0.74 *

Note. * refers to the square root of average variance extracted; LI = learner interface; CONT = content; PERS = personalization.

**Table 6:** Standardised loadings, average variance extracted, and construct reliability coefficients of the three-factor second-order model.

Construct and Items	Standardised Loading	Average Variance Extracted	Construct Reliability
LS		0.77	0.98
LI	0.81		
CONT	1.00		
PERS	0.81		
LI		0.64	0.98
LI1	0.85		
LI2	0.85		
LI3	0.82		
LI4	0.65		
CONT		0.61	0.99
CONT1	0.73		
CONT2	0.85		
CONT3	0.76		
CONT4	0.75		
CONT5	0.80		
CONT6	0.79		
PERS		0.54	0.97
PERS1	0.75		
PERS2	0.81		
PERS3	0.78		
PERS4	0.59		

Note. LI = learner interface; CONT = content; PERS = personalisation.

**Figure A1:**
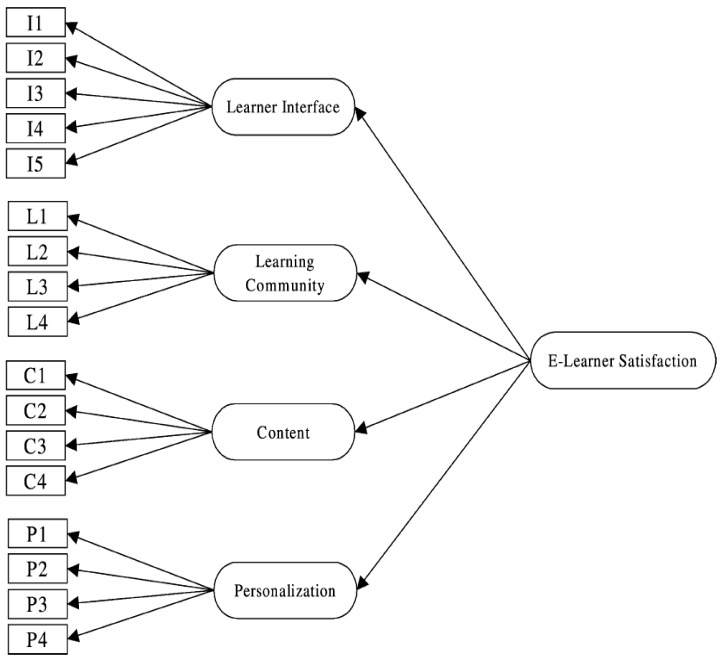
Wang’s (2003) proposed measurement model of the LSQ.

## Data Availability

Data available on request due to privacy and ethical restrictions.

## Appendix B

Learner Satisfaction Questionnaire (adapted)

(5-point Likert scale: 1 Strongly Disagree, 2 Disagree, 3 Neutral, 4 Agree, 5 Strongly Agree)

1. The system is easy to use (LI1).
2. The system is user-friendly (LI2).
3. The operation of the system is stable (LI3).
4. The system makes it easy for me to find the content I need (LI4).
5. The system provides up-to-date content (CONT1).
6. The system provides content that exactly fits my needs (CONT2).
7. The system provides sufficient content (CONT3).
8. The system provides useful content (CONT4).
9. The system enables me to learn the content I need (CONT5).
10. The system enables me to choose what I want to learn (PERS1).
11. The system enables me to control my learning progress (PERS2).
12. The system records my learning progress and performance (PERS3).
13. The system supports my learning * (CONT6).
14. The system recommends topics that reflects my learning progress * (PERS4).

Note: Items marked with “*” are new items. All items have been modified slightly (i.e., “you” has been changed to “I”; “e-learning system” has been changed to “system”; “your” has been changed to “my”).

## Appendix C

**Table A1:** Skewness and kurtosis values of LSQa items.

Variable	Kurtosis	Skewness
Q_1	0.7414892	−0.8999211
Q_2	1.8786178	−1.0495770
Q_3	1.0670180	−0.8986991
Q_4	−0.4323551	−0.4749700
Q_5	0.4405646	−0.5534692
Q_6	−0.6011248	−0.2631777
Q_7	−0.8418520	−0.2865288
Q_8	0.2607167	−0.6221956
Q_9	−0.4444183	−0.5042582
Q_10	−0.3667205	−0.3052960
Q_11	0.3347129	−0.6470117
Q_12	0.1587396	−0.5971343
Q_13	0.6893963	−0.6702294
Q_14	0.1228709	−0.7080154
